# Health Disparities in Staphylococcus aureus Transmission and Carriage in a Border Region of the United States Based on Cultural Differences in Social Relationships: Protocol for a Survey Study

**DOI:** 10.2196/14853

**Published:** 2019-09-27

**Authors:** Talima Pearson, Steven D Barger, Monica Lininger, Heidi Wayment, Crystal Hepp, Francisco Villa, Kara Tucker-Morgan, Shari Kyman, Melissa Cabrera, Kevin Hurtado, Ashley Menard, Kelly Fulbright, Colin Wood, Mimi Mbegbu, Yesenia Zambrano, Annette Fletcher, Sarah Medina-Rodriguez, Mark Manone, Amanda Aguirre, Trudie Milner, Robert T Trotter II

**Affiliations:** 1 Pathogen & Microbiome Institute Northern Arizona University Flagstaff, AZ United States; 2 Center for Health Equity Research Northern Arizona University Flagstaff, AZ United States; 3 Department of Psycological Sciences Northern Arizona University Flagstaff, AZ United States; 4 Department of Physical Therapy and Athletic Training Northern Arizona University Flagstaff, AZ United States; 5 School of Informatics, Computing, and Cyber Systems Northern Arizona University Flagstaff, AZ United States; 6 Northern Arizona University-Yuma Yuma, AZ United States; 7 Yuma Regional Medical Center Yuma, AZ United States; 8 Department of Geography, Planning and Recreation Northern Arizona University Flagstaff, AZ United States; 9 Regional Center for Border Health Somerton, AZ United States; 10 Department of Anthropology Northern Arizona University Flagstaff, AZ United States

**Keywords:** S aureus carriage, S aureus transmission, community acquired S aureus transmission, social determinants of health, social network analysis, border health, health disparities in minority communities

## Abstract

**Background:**

Health care–associated *Staphylococcus aureus* infections are declining but remain common. Conversely, rates of community-associated infections have not decreased because of the inadequacy of public health mechanisms to control transmission in a community setting. Our long-term goal is to use risk-based information from empirical socio-cultural-biological evidence of carriage and transmission to inform intervention strategies that reduce *S aureus* transmission in the community. Broad differences in social interactions because of cultural affiliation, travel, and residency patterns may impact *S aureus* carriage and transmission, either as risk or as protective factors.

**Objective:**

This study aims to (1) characterize *S aureus* carriage rates and compare circulating pathogen genotypes with those associated with disease isolated from local clinical specimens across resident groups and across Hispanic and non-Hispanic white ethnic groups and (2) evaluate social network relationships and social determinants of health-based risk factors for their impact on carriage and transmission of *S aureus*.

**Methods:**

We combine sociocultural survey approaches to population health sampling with *S aureus* carriage and pathogen genomic analysis to infer transmission patterns. Whole genome sequences of *S aureus* from community and clinical sampling will be phylogenetically compared to determine if strains that cause disease (clinical samples) are representative of community genotypes. Phylogenetic comparisons of strains collected from participants within social groups can indicate possible transmission within the group. We can therefore combine transmission data with social determinants of health variables (socioeconomic status, health history, etc) and social network variables (both egocentric and relational) to determine the extent to which social relationships are associated with *S aureus* transmission.

**Results:**

We conducted a first year pilot test and feasibility test of survey and biological data collection and analytic procedures based on the original funded design for this project (#NIH U54MD012388). That design resulted in survey data collection from 336 groups and 1337 individuals. The protocol, described below, is a revision based on data assessment, new findings for statistical power analyses, and refined data monitoring procedures.

**Conclusions:**

This study is designed to evaluate ethnic-specific prevalence of *S aureus* carriage in a US border community. The study will also examine the extent to which kin and nonkin social relationships are concordant with carriage prevalence in social groups. Genetic analysis of *S aureus* strains will further distinguish putative transmission pathways across social relationship contexts and inform our understanding of the correspondence of *S aureus* reservoirs across clinical and community settings. Basic community-engaged nonprobabilistic sampling procedures provide a rigorous framework for completion of this 5-year study of the social and cultural parameters of *S aureus* carriage and transmission.

## Introduction

### Overview

*Staphylococcus aureus* is a Gram-positive bacterium that lives in close association with humans as both a commensal and pathogen [1]. Methicillin-resistant *S aureus* (MRSA) and methicillin-sensitive *S aureus* (MSSA) infections have historically been considered a health care–acquired (HA) phenomenon. However, community-acquired (CA) MRSA and MSSA now represent the most common cause of skin and soft tissue infections (SSTIs) presenting to US emergency departments [2]. In 2017, an estimated 120,000 invasive MRSA infections occurred in the United States, with 20,000 associated deaths [3], whereas MSSA infections sometimes outnumber MRSA infections 3 to 1 [4]. Invasive MRSA infections in health care settings have been declining, but community MSSA infections are increasing slightly [3,5].

A number of significant challenges exist for controlling *S aureus* transmission in general populations. Even though rates of HA MRSA infections have declined since 2005 and 2007, they remain unacceptably high, and rates of CA MRSA have not decreased over this period [3,5-12]. About one-third of the healthy US population is thought to be asymptomatic carriers of *S aureus* with 1.5% being carriers of MRSA [13]. These carriers form an important community-based reservoir for MSSA and MRSA infections. Colonization and infection rates have not been thoroughly studied for Hispanics; however, there is evidence that MRSA colonization is lower for Hispanics than for non-Hispanic whites [14]. Although Hispanics have a lower risk of SSTIs than non-Hispanic whites [15], isolates recovered from SSTIs are more likely to be MRSA [15], which are associated with increased risk of MRSA infection [16]. However, local conditions impacting community health may modify these associations.

Transmission of *S aureus* occurs through contact with colonized individuals, animals, and contaminated surfaces. About a third of all Americans are thought to be asymptomatic carriers of *S aureus*, and about 1.5% of the general US population are carriers for MRSA [13]. Although the throat and nares are most often colonized, other warm and moist body sites such as the axillae, anus, vagina, and perineum are also frequently colonized [17-20]. *S aureus* can survive in the environment, depending on conditions, for over 90 days [21]. Importantly, hands can also be transiently colonized and can transfer the bacteria to other body areas and other individuals [4]. Persons colonized with *S aureus* are at risk of infecting themselves, although most do not develop clinical disease [4]. In a hospital setting, approximately 30% of MRSA colonized patients developed subsequent SSTIs [22].

Intervention programs have reduced MRSA in health care institutions, but a more thorough understanding of transmission networks is required for efficient and targeted mitigation of MRSA and MSSA in community settings. Comprehensive efforts to decolonize people and decontaminate their environment reduced but did not eliminate long-term carriage and recurrence of disease. For example, 49% of children infected with CA-*S aureus* were reinfected within 6 months of decolonization [23]. How are these patients becoming recolonized and reinfected? Unlike the more controlled hospital environment, transmission chains for CA-MRSA and MSSA are more complex, and sources may be more difficult to identify. On a limited basis, search and destroy principles have been extended into patient households and have involved decontaminating household surfaces and screening/treating family members and even pets [17,24]. In 1 study, after index patients were decolonized, 72% were reinfected after 12 months, and even when all household members were additionally decolonized, 52% of index cases were reinfected [25]. Importantly, social contacts within and outside the immediate household are likely to play a role in the transmission of [26].

The Yuma County Public Health District has identified *S aureus* (MRSA and MSSA) as a top public health priority in this border health district, based on community engaged environmental scans conducted by Northern Arizona University’s (NAU’s) Center for Health Equity Research in collaboration with the NAU-Yuma branch campus [27].

Study site is Yuma County, Arizona. It has an overall population of 212,128 (2018 estimate). The race and ethnicity breakdown of the resident population is 63.9% Hispanic, 30.8% white non-Hispanic, 2.7% African American, and 2.3% Native American [28]. The majority of the population of Yuma County is concentrated in the southwestern corner near the city of Yuma (about 50% of the county’s population). The US Department of Labor’s Bureau of Labor Statistics ranked the unemployment rate of 389 metropolitan areas and found that Yuma has the third highest unemployment rate in the country, at 12.3% [29]. Yuma is identified as a medically underserved community. Nearby cities in the Imperial Valley of California (Calexico, El Centro, and Imperial) have a combined population of approximately 102,000 of which more than 86% are Hispanic. This entire region serves as the catchment area of the Yuma Regional Medical Center (YRMC). Yuma sustains a large agricultural labor force and frequent cross-border interactions with migrant farm worker populations from Mexico. In addition, an estimated 90,000 “winter visitors/residents” live in the area during the winter and early spring months. As a consequence of its large cross-border agricultural worker population, the ethnic make-up of the residential community, and seasonal population dynamics, the area surrounding Yuma, Arizona, provides a unique setting to investigate the social and biological parameters of *S aureus* carriage and transmission.

### Research Questions

We are comparing community and clinical samples to examine ethnic-based carriage disparities. We will also determine whether *S aureus* transmission is associated with social network attributes and culturally based relations within each naturally occurring social group in the sample. The ability to compare MSSA and MRSA carriage and transmission within and between these groups presents a significant opportunity to examine the complexity of any carriage or infection-related health disparity. It also has the potential to establish improved models for intervention in culturally complex populations. Our research methods combine sociocultural survey data collection with population health, biological sampling, and genomic analysis. We will use a synthesis of approaches (asking participants questions designed to quantify contact within their naturally occurring social group and comparing the evolutionary relatedness of *S aureus* positive samples to infer transmission) to determine the extent to which social relationship characteristics are associated with *S aureus* transmission.

Specific aim 1 is as follows: We will determine if there is an ethnic and residency-based *S aureus* carriage disparity in Yuma, Arizona. We will also compare circulating pathogen genotypes isolated from asymptomatic community members with those associated with disease isolated from local clinical specimens.

Hypotheses are as follows: (1a) Ethnic-based disparities in *S aureus* infections extend to carriage in the populations of 2 ethnic groups (Hispanic and non-Hispanic white) in Yuma, Arizona. We will also test the null hypothesis (1b) that circulating *S aureus* genotypes do not differ among the ethnic groups and thus do not account for any observed disparities. To do this, we will obtain a community sample of *S aureus* by collecting samples from people recruited at multiple community sites and events. The genome sequences from this community sample will be compared with sequences from clinical *S aureus* samples collected from patients at local health care providers.

Variables are as follows: We will collect nasal, oropharyngeal, and hand swabs to test for the presence of *S aureus* and thus determine the community carriage proportions across ethnicities and by part-time versus full-time resident groups. We will compare the proportions of community *S aureus* carriage to determine if these stratifications present a disparity. Positive samples will be sequenced and phylogenetically compared with genomes from clinical infection isolates collected at the YRMC. This will determine concordance, if any, between clinical pathogen genotypes and strains carried within the general population. In addition, we will determine if clinical and community strains are clustered by ethnicity and thus could partially explain discrepant likelihoods of *S aureus* infections.

Specific aim 2 is as follows: We will evaluate social network relationships and social determinants of health-based risk factors for their impact on carriage and transmission of *S aureus*.

Our hypothesis is as follows: (2a) Differences in the types and intensity of social contact behavior within and between the populations will result in differential *S aureus* transmission within social groups. While enrolling participants, we will target a variety of naturally occurring social groups (family, friends, and coworkers). We will determine the extent to which social relationships result in *S aureus* transmission by (1) asking participants questions designed to quantify direct and indirect physical contact within a group and (2) assessing the evolutionary relationships (transmission) among positive *S aureus* samples through phylogenetic analysis of whole genome sequences to confirm or refute putative transmission.

## Methods

### Baseline/Pilot and Feasibility Studies

The original community sampling design for the project was to collect both social and biological data over a 4-month window each year by interviewing individuals in naturally occurring groups and collecting biological specimens from those individuals, comprising 367 social groups (family/friendship clusters) in residential and public settings around Yuma, Arizona (Yuma Somerton, San Luis, and Rio Colorado), and nearby communities in the southernmost parts of the Imperial valley, California (Calexico, El Centro, and Imperial). Individuals were consented and enrolled into the study for biological sampling and assessment of social relationships. *S aureus* samples from the nose, throat, and hand of each participant were collected and are being processed for whole genome sequencing. The proportion of positive samples from individuals of each ethnicity are being compared with the proportional composition of each ethnicity in the naturally occurring social groups sampled. All *S aureus* isolates are undergoing whole genome sequencing and phylogenomic analysis to identify clustering by ethnicity, residential status, or geography. Clinical samples (residual diagnostic specimens) are being collected throughout the year to determine temporal patterns and determine if clinical isolates are representative of the genomic diversity present in the community samples. Data from the deidentified clinical samples include diabetes (yes/no), age, gender, ethnicity, and state of residency. We are using social determinants of health (education and wealth), individual-level social integration, and group-level social network variables (both egocentric and relational) to determine the extent to which social relationships can explain or contribute to *S aureus* transmission. Social relationships and contact are being determined by asking participants questions designed to quantify physical contact within a social group. By comparing the evolutionary relatedness of *S aureus* genomes with the measured or estimated level of contact among participants, we will be able to determine social contact variables most likely linked to transmission.

### Sampling Approach

In the year 1 baseline and feasibility study, we used a stratified purposive sampling approach [30-33] to capture naturally occurring social groups at both private and public venues in our study area. Recruitment consisted of inviting group members to participate in this study, assuming that their interactions represent a variety of direct and indirect contact relationships (close family and friends will interact more and be more likely to transmit *S aureus* compared with distant family members or acquaintances). Participant inclusion criteria was full-time or part-time residence in Yuma for at least 1 member of the social group. All members of the recruited social group were invited to participate in the project. Analysis of carriage by ethnicity will incorporate group clustering.

Given the potential sensitivity of human genetics research, informed consent is designed to clearly convey that no human genetic material will be maintained or analyzed. Each consented individual (or in the case of children; child assent and parental consent) was asked to complete the survey containing social and demographic information and to be swabbed (anterior nares, throat, and dominant hand) to detect both *S aureus* colonization. The genomes of *S aureus* cultures are being sequenced to determine strains and phylogenetic relationships to infer transmission within naturally occurring social groups. In addition, these genomes can be compared with *S aureus* genomes from clinical isolates collected across the same time period from the YRMC. As the primary catchment area of YRMC includes communities in Arizona and California that are targeted in this study, comparison of clinical and community genomes will allow us to determine if clinical isolates are representative of community isolates and if prevalent clinical isolates change over time.

Comparisons of carriage across ethnic lines will enable us to determine if there is a *S aureus* carriage disparity in the general population. Subsequent whole genome sequencing and phylogenetic analysis will allow us to determine if clinical *S aureus* strains are representative of the diversity of community strains and not because of the emergence of a few highly fit lineages. Phylogenetic analyses will also be used to exclude the possibility that *S aureus* populations differ along ethnic and residential groups, thus eliminating pathogen genotype as a partial explanation for any carriage or infection disparity. In addition, phylogenetic analyses will be used to identify possible transmission events within a social group. From all community participants, we have collected demographic, economic, and social interaction data. These data permit evaluation of the relative importance of social and demographic colonization and transmission determinants in this diverse border community. Using binary *S aureus* carriage (yes/no) as the outcome, we will use logistic regression to determine the strongest indicators of colonization and transmission in the community.

We are also evaluating social network– and social determinants–based risk factors for transmission of *S aureus*. While enrolling participants, we targeted a variety of social groups (family, friends, and coworkers). Whole genome sequencing and phylogenetic analyses of resulting *S aureus* genomes will allow us to determine if transmission has occurred between members of a social group. By asking participants questions designed to quantify direct and indirect physical contact with each member of the social group, we will be able to determine the extent to which social relationships are associated with *S aureus* transmission.

Each year, sampling will take place over the same 4-month period in residences, businesses, and at multiple public locations within the main cities of Yuma County, Arizona.

### Recruitment and Sampling Processes

After consultation with community partners and local residents, we designed the recruitment and sampling process to take advantage of available community resources. The recruiters were identified and trained (as a cohort) as part of a required undergraduate social work research methods class. A total of 28 bilingual recruiters were provided with basic research design training, ethics training (Collaborative Institutional  *Training* Initiative and face to face), recruitment role playing, technology (computer-assisted data entry) training, and biosample collection procedures, with monitoring and follow-up by course instructors and project staff. This process provided the opportunity to target specific sized groups, ethnic makeup, and geographical coverage of sampling. Table 1 identifies the targeted social group size, the number of groups of each size to be targeted, and the breakdown of groups to be recruited by each recruiter/consenter/interviewer. CA- *S aureus* infections peak after the hottest months of the year [34-36]; however, neither agricultural workers nor seasonal visitors are in Yuma at this time. Consequently, the time frame for our sampling was during late winter. Our anticipated sample size is not reliant on sampling when carriage rate is predicted to be highest.

Each recruiter was assigned to recruit a total of 12 social groups, stratified as one-third non-Hispanic white groups and two-third Hispanic groups. The total targeted individual recruitment, based on group size, was 1512. Both group and individual sample sizes had appropriate power, based on an *a priori* power analyses to detect differences of 15% in carriage rate (if one truly exists) and important predictor variables for transmission.

**Table 1:** Targeted sampling framework for social network and community biosamples.

Variable	Social group size									Total
	2	3	4	5	6	7	8	9		
Number of groups per recruiter	3	3	1	1	1	1	1	1	12	
Total groups targeted	84	84	28	28	28	28	28	28	336	
Total individuals targeted	168	252	112	140	168	196	224	252	1512	

## Results

### Baseline Effort

The recruiters were able to effectively recruit targeted groups in both residential (mostly family, friends, and neighbors), and public locations (work, school, and public access areas). A total of 2 of the interviewers were not able to complete any viable interviews. From YRMC, we collected 660 clinical isolates associated with SSTIs.

We will characterize *S aureus* infection and carriage rates and compare circulating pathogen genotypes with those associated with disease isolated from local clinical specimens across full-time and part-time resident groups and across Hispanic and non-Hispanic white ethnic groups. Positive samples will be sequenced and compared through phylogenetic analyses with genomes from infection specimens collected at the YRMC to determine if the diversity of the clinical pathogen genotypes is representative of the diversity of strains carried within the general population. In addition, we will determine if clinical and community strains are clustered by ethnicity and thus could partially explain discrepant likelihoods of *S aureus* infections.

### Quality Control Processes

The baseline/pilot protocol incorporated a systematic data quality control mechanism including (1) interviewer training and reinforcement of fidelity to the data collection protocol and (2) review of geolocation to assess the spatial distribution of recruited groups and individuals, quality of upload and data integrity of transmitted data, as well as group and one-on-one follow-up on protocol and data integrity review at the end of the field cycle for both survey and biological data collection. The systematic review identified several problems that have subsequently been addressed through quality control measures. A total of 2 of the interviewers provided suspect and unverifiable data, which was subsequently removed from the dataset. A total of 2 other interviewers failed to upload a revised version of the data collection instrument. Those datasets were tagged and cleaned/noted for appropriate analysis. Another interviewer collected answers to each question but deviated from the protocol by entering responses for the participant. As this was not approved, those data were eliminated from the overall dataset. After excluding cases (n=70) from the above interviewers, we recruited a total of 335 groups (group size: 2=89, 3=79, 4=39, 5=33, 6=24, 7=24, 8=24, and 9=23), which included 1267 individuals (Table 2).

Through our postdata collection evaluation, we identified ways in which our interviewer training, the data collection technology, sampling logistics, and data quality monitoring protocols could be improved. Data collection occurred using the Survey123 app (ArcGIS) on Samsung Galaxy Tab A 7.0 tablets. Some respondents had to be assisted with the touchscreen nature of the survey. The geographical location of the tablet at the time of the survey was automatically recorded, but sometimes failed, presumably because of an inability to obtain satellite global positioning system location. User input of geolocation of their residence and work was also inconsistent as these questions could not be set as required by the software and were often inadvertently skipped. In addition, the survey app sometime crashed. No data were lost, and restarting the software returned the respondent to the place where they left off but was an inconvenience. The integrated survey123 barcode scanner was slow and ineffective. We used a separate barcode scanner to read the group identity document (ID), which was then be copied and pasted into the survey to link data from individuals within a group as well as link survey and biological data. Due to this extra step, surveyors often manually entered group ID information that sometimes introduced errors. For some respondents, there was confusion over the letter designation scheme we used to identify individuals within each group, causing erroneous data on individual relationships within each group. On the basis of this feedback and review of data, future interviews will be administered in paper and pencil format. Such a change allows group data to be collected in a timelier manner (no need to wait for an available tablet), requires less assistance from interview staff, eliminates software glitches, and provides a hard copy backup once data are entered. Additional confusion can be greatly reduced in the new data collection protocol by improved explanations, training, and formatting of the revised instrument. We used the protocols established by Biemer [37] to identify additional ways in which to ensure and improve data accuracy, credibility, usability, relevance, accessibility, and completeness.

The quality control screening excluded a substantial proportion of year 1 data. This screening included checks to ensure the correct number of respondents in each group as well as checks for within-group response consistency and social relationship fidelity (eg, correct self-identification and mutually consistent kin and spouse identification). These exclusions ensure social group integrity and are tracked as per STROBE guidelines [37]. There were 168 groups encompassing 633 individual respondents after this quality control screening.

**Table 2:** Baseline demographic, economic, and social characteristics of Yuma pilot year 1 data.

Participant characteristic		Value^a^
Age^b^ (years), mean (SD)		28.1 (14.2)
**Sex, n (%)**		
	Female	384 (60.7)
	Male	289 (39.3)
**Race, n (%)**		
	White	330 (52.1)
	Black	37 (5.9)
	No preferred race	139 (22.0)
	Other race only	70 (11.1)
	Multiracial	57 (9.0)
**Ethnicity, n (%)**		
	Hispanic	172 (27.2)
	Non-Hispanic	461 (72.8)
**Educational level, n (%)**		
	Less than high school	305 (48.2)
	High school	114 (18.0)
	Some college	142 (22.4)
	College graduate or higher	72 (11.4)
**Employment status, n (%)**		
	Employed	350 (55.3)
	Retired	11 (1.7)
	Not currently working	26 (4.1)
	Homemaker	17 (2.7)
	Student	164 (25.9)
	Missing	65 (10.3)
**Home tenure, n (%)**		
	Own home	286 (45.2)
	Rent/other arrangement	347 (54.8)
**Self-rated health, n (%)**		
	Poor/fair	70 (11.1)
	Good	258 (40.8)
	Very good	185 (29.2)
	Excellent	79 (12.5)
	Missing	41 (6.5)
**Social group size, n (%)**		
	2	102 (16.1)
	3	162 (25.6)
	4	64 (10.1)
	5	80 (12.6)
	6	60 (9.5)
	7	49 (7.7)
	8	80 (12.6)
	9	36 (5.7)

^a^Some percentages do not sum to 100 because of rounding.

^b^One value of age was missing.

### Year 2: Revised Data Collection Protocol

The review processes resulted in improvements for year 2 data collection efforts. We now describe our revised protocol that is designed to eliminate the previously discovered threats to data integrity and provide a more transparent and traceable data collection process.

#### Recruitment and Consenting

We will continue to use “culturally congruent recruiters” to manage the quality of interview and refusal rate. Working with our community partners, we are implementing a broader community matched configuration for recruitment for our consenters and interviewers. To the extent possible, the demographic characteristics (age, gender, ethnicity, and language preference) of the interview teams will match the demographic characteristics of the target population.

#### Recruiter Training

Recruiters receive a combination of didactic, hands-on, and role playing training (approximately 8 hours), followed by a minimum of 3 supervised field-based data collection sessions (approximately 8 hours), with direct feedback on all aspects of recruitment, data collection, and data transmission. All data collection sessions are then monitored for a minimum of 1 month, with periodic checks and problem solving debriefing each week. The didactic training comprises an overview of the project (both theory and hypotheses), introduction to recruiting principles and scripts, questionnaire details, biological sampling, and ethical (institutional review board) considerations. The hands-on elements include practice delivering scripts and answering anticipated participant questions. This element is followed by role playing to practice recruiting, informed consent, biological sample collection, and questionnaire delivery using other trainees and staff to work through the data collection process. Once the principal investigator and investigators are satisfied with data collection competency, the recruiters take part in at least 3 separate recruitment events with procedural review before each event and full debriefings after each group is recruited and provides data. All data are collected by 2-person recruiter teams to preserve data collection integrity. Weekly debriefings to resolve any unanticipated problems are conducted by supervising staff.

#### Sample Size and Sampling Plan

We will continue to use a stratified purposive sampling approach to capture socially associated groups at both private and public events and venues in Yuma County. Each year, we aim to recruit 370 groups with a target of 243 Hispanics and 122 non-Hispanic whites. This will give us the power to detect a 15% difference in carriage rates. We will enroll naturally occurring social groups that will likely include family, friendship, work, and neighbor relationships. Such sampling will thus produce a wide variety of types of social contacts of varying closeness and strength.

#### Inclusion and Exclusion Criteria

Our recruitment process is aimed at capturing local migrant laborers as well as part-time winter residents but excluding groups consisting of only tourists as they are less likely to reflect local *S aureus* carriage rates but may contribute to transmission within a social group. The use of a proxy to respond to the survey for nonliterate children as well as the person ID for the proxy within the group will be recorded. Any individual younger than 6 months will be excluded. Our aim is to sample a representative section of the community. We will, therefore, not specifically target at-risk groups to avoid biasing our estimation of carriage within the general population. In addition, our projected sample size is based on a power analysis using the carriage rate of the general population and should provide a sufficient number of positive samples to address our questions without targeting at-risk groups.

On the basis of data captured, cleaned, and preliminarily analyzed during the pilot (year 1), the number of groups to be recruited in different venues is listed in Table 3.

Each consented individual will be asked to respond to the *S aureus* data collection instrument and will be swabbed (anterior nares, throat, and hand) to detect both MSSA and MRSA colonization. The *S aureus* cultures will go through whole genome sequencing to determine phylogenetic relationships. Such analyses will enable us to identify cases of likely transmission within a group. Genomic comparisons to be compared with *S aureus* MSSA and MRSA clinical isolates collected from YRMC will allow us to determine if the clinical strains are a representative subset of those circulating in the community.

**Table 3:** Year 2 targeted group recruitment for Yuma County.

City	Public spaces, n			Public events, n			Private spaces, n			Total groups (N=370)
	A^a^ (n=56)	H^b^ (n=112)	A (n=56)		H (n=112)	A (n=31)		H (n=62)		
Yuma	50	75	50		75	26		38	314	
Somerton	2	4	2		4	2		4	18	
San Luis	4	9	4		9	4		8	38	

^a^A: Non-Hispanic white groups recruited.

^b^H: Hispanic groups recruited.

#### Variables

This paper and pencil survey consist of 33 items with an additional 7 items on relationship for each person within the group. We collect basic demographic information, health status, alcohol use, level of contact (physical and social), and identification of social relationships within the group. We include a general measure of health as there is evidence that *S aureus,* colonization is associated with health disparity indicators such as poor health status [38-41]. We also assess educational attainment and home ownership given that *S aureus* colonization may also be associated with socioeconomic status [14]. We will, therefore, use well-tested variables that permit partitioning of the relative potency of ethnic versus socioeconomic determinants of colonization [42]. The social integration measures predict morbidity and mortality [43,44] including increased susceptibility to infectious diseases [45]. Some of these factors have previously been shown to be important for determining likelihood of infection and will be used with ethnicity to determine the best predictors of colonization. The source variables include demographic questions from the US National Health Interview Survey (NHIS; gender, age, ethnicity, income, education, and employment); general health, including history of infections and antibiotic use; and health care access, including location of health care services; questions identified as risk factors for CA-MRSA transmission risk (number of people in household and contact with animals). The US NHIS items have validated Spanish language translations [46]. We also assess perceived strength of physical contacts between members of the social group with contact defined as either direct physical contact that would increase the probability of *S aureus* transmission, or indirect physical contact through shared contact items or conditions [34]. Each individual in a social group is asked to complete background information about themselves and afterward answer 7 questions about each member of their group regarding the nature and intensity of their contacts (ie, type of relationship: romantic partner, parent, sibling, friend, coworker, etc, closeness of relationship, amount of physical contact, frequency of social contact, shared meals and living space, and shared pets). The composite network data will provide data on individual connectivity. Macro-level social networks will be assessed with the Social Network Index [45], which is predictive of susceptibility to infection [45] and captures social integration features associated with mortality [47]. Added to this index are other social contact questions assessed in US public health surveys that are also predictive of hard health end points [43,48]. The entire data collection instrument has been translated into Spanish following World Health Organization–recommended protocols that include translation-back translation and cognitive debriefing [49,50]. We also checked the linguistic phrasing of the instruments for local vernacular.

The social network and social determinants data collection instrument will be integrated with biological data instrumentation such that data are collected concurrently.

#### Biological Data Collection

After recruitment and consent, participants are directed to provide biological samples before answering survey questions on the data collection instrument. This workflow is designed to minimize the possibility of cross-contamination of participant and researcher microbes. Sample collection kits are prepackaged and prelabeled such that a gallon-sized Ziploc bag is labeled for a group and contains quart-sized Ziploc bags labeled for each individual within a group. The “group” bag also contains gift cards (as incentives) and name-tag stickers written with “A,” “B,” “C,” etc, depending on the number of people within the group. These name tags and naming scheme allow for the maintenance of anonymity as respondents answer questions about their relationships within a group. Each “individual” bag (all within the “group” bag) contains 6 prelabeled BBL CultureSwabs. Swab labels include participant IDs as well as the body site to be sampled. Prelabeling and packaging of culture swabs is done aseptically (using nitrile gloves followed by a wipe down with ethanol) to prevent contamination with microbes from the preparing laboratorian. Nesting the “individual” bags within the “group” bag also eliminates the need for the field researchers to handle swabs as individual participants handle and open their own bag.

After opening the group bag, researchers pass out the prefilled name tags while explaining that the letter on the name tag is the identifier for each person within the group. “Individual” bags are then distributed, and respondents are instructed to remove a specific color-coded prelabeled swab. All participants are then guided through swab handling and swabbing methods. To further ensure consistent sampling, the researcher counts off 20 seconds while instructing participants to continue swabbing and turning the swab for each body site (palm, nose, and throat). The palm of only 1 hand is swabbed, but for the nose, both nares are swabbed for 10 seconds each. After each swab, the researcher holds out the empty “group” bag for participants to drop the used swab into. When all swabs are completed and collected, researchers seal the “group” bag and push on both ends of the bag to ensure that all swabs are completely closed within the bag. Bags containing used swabs are stored in a cool place for as short of a time period as possible before placing them on ice or in a refrigerator at approximately 4°C. After sample collection, participants are provided with the survey, which typically takes approximately 12 to 15 min for the group to complete, based on timed interviews in the field.

Biological samples are transported to our laboratory on the NAU-Yuma campus where the swabs are stored at 4°C before streaking on CHROMagar Staph aureus plates. These plates are specific to Gram-positive organisms and contain chromagens that turn *S aureus* colonies fuchsia for easy and accurate identification. Plates are read after 24 to 26 hours of incubation at 37°C. For samples that are determined to be positive for *S aureus*, selected growth is scraped off the plate and placed into cryogenic vials containing 20% glycerol for long-term storage at –70°C for subsequent culturing and isolation, DNA extraction, and whole genome sequencing at the NAU flagstaff campus.

#### Quality Control Processes

Given the success and importance of the systematic data quality control mechanisms implemented during the baseline/pilot data collection, we have instituted a more comprehensive protocol for ensuring data quality. Surveyor training now includes additional discussions of questionnaire content, role-playing, and supervised data collection in the field. Immediate review of surveys in the field now allows for the identification of common errors and a discussion with the surveyors on mitigation of such errors.

## Discussion

Our study will examine carriage, pathogen genotypes, clinical versus community strains of *S aureus* and the impact of social relationships and shared physical environments on carriage. Our study will also utilize genomic analysis to infer transmission patterns across social and geographic environments and distinguish between antibiotic resistant and susceptible *S aureus* subtypes. These complementary approaches should provide a better understanding of *S aureus* transmission and inform more robust infectious disease intervention and prevention strategies.

## Appendix

Multimedia Appendix 1 Peer-review reports from NIH.

## Abbreviations

CA: community-acquired
HA: health care–acquired
ID: identity document
MRSA: methicillin-resistant *Staphylococcus aureus*
MSSA: methicillin-sensitive *Staphylococcus aureus*
NAU: Northern Arizona University
NHIS: National Health Interview Survey
SSTI: skin and soft tissue infection
YRMC: Yuma Regional Medical Center
