# Supplementary material for: Health Disparities in Staphylococcus aureus Transmission and Carriage in a Border Region of the United States Based on Cultural Differences in Social Relationships: Protocol for a Survey Study
Source: JMIR Res Protoc. 2019 Sep 27;8(9):e14853. doi: 10.2196/14853 (PMC6789426; doi:10.2196/14853)
Supplement: Multimedia Appendix 1 [file resprot_v8i9e14853_app1.pdf]

U54 REVIEWER CONCERNS – Research Project #1 (Pearson)

Research Project 1, Do Pathogen Genotypes, Carriage, and Social Network Differences Lead to Health Disparities in MRSA/MSSA Infections?, proposes to evaluate the disparity of *S. aureus* carriage/infection among Hispanics who have a lower risk of infection, versus Caucasian and American Indians who have a higher risk of infection. The project proposes to use genomic-based methods to characterize methicillin sensitive *S. aureus* (MSSA) and methicillin resistant *S. aureus (*MRSA) infection and carriage rates and compare circulating pathogen genotypes with those associated with disease isolated from local clinical specimens across full-time and part-time resident groups, and across Hispanic and Non-Hispanic White ethnic groups. Risk of infection transmission will be determined using social determinants of health and social network variables. This community approach project has significance to enhance the understanding of the disparities and potentially lead to new interventions.
Linking genome-based epidemiology to social contact is a novel approach. The project is driven by community needs assessment showing MRSA is one of the top five health issues in the County. The community environment is excellent for this type of study. A concern is the weak premise of the hypothesis that ethnic-based disparities in MSSA/MRSA infections extend to MRSA/MSSA carriage in the populations of two ethnic groups in Yuma, Arizona. Among the panel some felt that a major weakness includes the potential selection bias in the community, missing the population that do not go to public events, although recruitment during seasonal work will reduce bias. In addition, adjustment for social networks or clusters of participants within families is also not considered. Confounders, such as age or sex variable or co-morbidities, lack discussion and are also weaknesses. Clinician expertise is not sufficiently represented. In general, potential difficulties are not discussed.
Response 1: One of the hypotheses of SA#1 is that ethnic-based disparities in infection extend to carriage in the Yuma populations. While the premise for this is based on a small number of studies that addressed infection and carriage in minority populations, they all show that Hispanic Americans are more likely to be colonized by *S. aureus*, but less likely to have infections. Conversely, Hispanics born in Mexico are less likely to be colonized. We also have data from Northern Arizona that show a disparity in MSSA and MRSA infections among three ethnic groups (Native American, Hispanic, and Anglo). While it is clear that MSSA/MRSA infections are a big problem in Yuma, we do not yet know how the rates of infection and colonization, stratified by the different populations, compare. One of the initial rationales for the disparity hypothesis is the inclusion of a social network perspective within the overall transmission hypotheses. There is excellent evidence in the literature that the "contact" networks of Hispanics and Anglos differ on the basis of cultural differences in the shapes of family, extended family, and friendship networks. Testing this hypothesis can lead to the understanding of the social mechanisms impacting both carriage and infection. While it is likely that we will find disparities among our subject populations, the success of this work does not depend on our finding any disparities among our subject populations. Knowledge of the relationships between carriage and infection rates, circulating genotypes, and the impact of social behavior will greatly impact our understanding of *S. aureus* transmission and help address disparities that have been well documented elsewhere in the state and country (even if such disparities are not evident in our populations).
Response 2: We intend to minimize selection bias by holding at least 25 sampling events at 15 different locations. These locations will not be limited to "public events", but will include private commercial sites (e.g. malls, shopping centers, grocery stores) in order to minimize such bias and include a wide representation of the community. We will also use the extensive expertise of our regional partners (Regional Center for Border Health, Campesinos sin Fronteras, Yuma County Health District, Yuma Regional Medical Center)

in reaching “hard to reach” populations in Yuma county, using the type of “targeted sampling” frameworks that are commonly used in these types of studies. Each of these partners has extensive outreach programs and conducts or accommodates sampling of all of the relevant populations (Anglo, Hispanic, resident, snowbird) throughout the county. Our Center for Health Equities Research, and our core staff have created a Yuma community advisory group that has significant exptertise in identifying both locations and timing for sampling the populations in Yuma that will lessen response bias in our data. The first 3 months of the project will include designing and testing the final sampling frame for the research, within the context of meeting all of the scientific goals of the project. Sampling will also be conducted throughout the week to reduce any temporal fluctuations. We recognize that our methods will miss certain people, such as those in short and long-term healthcare facilities, however transmission is relatively well-understood and controlled in these settings and our aim here to focus specifically on community based transmission.
Response 3:. Our goal is to use empirical evidence and reported levels of contact to evaluate the likelihood of transmission. We anticipate that the reported relationship type (e.g. friends, brother/sister or mother/son) will correlate with levels of contact, and in the final analysis we may produce a weighted or adjusted level of contact that allows for a more refined test of this hypothesis, but we feel that a priori adjustments based on relationship type might lead to a bias and less accuracy, given the variation in contact levels within specific relationship types.
Response 4: We intend to adjust our model for possible confounders such as age, sex, and co-morbidities to more clearly determine the effects of such independent variables on the dependent variable (transmission). These will collected by the data collection instrument provided to each participant.
Response 5: We will be working closely with infectious disease specialists at both the YRMC and RCBH. In particular, Dr. P. Bhatt MD, (Internal Medicine) who is the Medical Director for RCBH/San Luis Walk In Clinic. We also work closely with Dr. J. Terriquez at the Flagstaff Medical Center on a separate project. We therefore have access to such expertise in Yuma and in Flagstaff. This project is not designed to address transmission in a healthcare setting, however we do have multiple contacts with such expertise that can help guide us as we identify factors that are important for carriage and transmission in the community.

Core/Project: Research Project 1
Title: Do Pathogen Genotypes, Carriage, and Social Network Differences Lead to Health Disparities in MRSA/MSSA Infections?
Lead/Co-Lead: Pearson/Trotter
Component Impact Score: 24

<u>Reviewer #1</u>
Significance: 3
Investigators: 2
Innovation: 4
Approach: 4
Environment: 2

1. **Significance**

This project is focused on understanding mechanisms of the disparity in MSRA/MSSA infections among Hispanics and whites living in and around the Yuma Arizona areas. The ultimate objective of this proposal is that enhanced understanding of the disparity may lead to new intervention strategies.

Strengths

- Ethnic differences in MRSA infections rates are higher with Native Americans and

lower with Hispanics than the general population. The basis of this disparity is unknown and data on whether clinical-based differences (infection rates and pathogen genotype) among ethnic groups extend into their communities in the form of non-symptomatic MSSA/MRSA carriage are lacking.

- The applicant proposes to identify ethnic differences in MRSA and MSSA carriage rates and the role of social behaviors in transmission. This project is focused on a community setting.

Weaknesses

- More information on the impact of community associated MRSA/MSSA infections in the local targeted community would have helped to place the significance of this study in context.
  Response 6: See Response 1.

2. Investigator(s)

Strengths

- Investigators are well suited to perform these studies

Weaknesses

- Need contribution from infectious disease specialist on this project.

Response 7: See Response 5 about our inclusion and ongoing collaborations with infectious disease specialists both in Flagstaff and in Yuma

3. Innovation

Strengths

- Approaches for limiting new and recurrent MRSA infections are proven strategies in a hospital environment but have had limited success in a community environment. This proposal will use genomic based phylogenetic hypotheses overlaid with defined and quantified social contact information to better understand how social interactions impact transmission and ultimately guide intervention models to limit transmission.

Weaknesses

- While this research represents the union of bacterial pathogen genome-based epidemiology with social contact analysis, social contact data have been useful in the control of sexually transmitted disease, which limits the innovative potential of this approach. However, it is noted that this approach could prove effective.

Response 8: We agree. These individual approaches are highly validated and have been highly effective for controlling different sexually transmitted diseases even though they have not been combined to address other bacterial-based diseases. Part of the innovation here is in the technological (translational) transfer of evidence based techniques to a new disease venue. One note that is relevant, is that these contact tracing network intervention designs have primarily been used in both hard to reach and heavily stigmatized populations (both population sigma and disease stigma). Since MRSA/MSSA are serious public health problems, but are not heavily stigmatized, this may be in important opportunity to determine whether or not the successful model for contact tracing and intervention is, minimally, as effective, more effective, or less effective in relation to non-stigmatized diseases.

4. Approach

Strengths

- Applicant proposes to recruit and consent over a four months' window each year, individuals from 367 social groups (family/friendship clusters) in public settings and attending public events in Yuma and enroll them into the study for biological sampling and administration of the social instrument.

Weaknesses

- Longer sampling periods will improve project Approach.

  Response 9: Under different circumstances, longer sampling periods would indeed improve the likelihood of capturing the temporal dynamics of *S. aureus* carriage and transmission. However in this study, we are limited to a four month window because major portions of our subject populations (snowbirds and agricultural workers) are predominantly present in Yuma during the winter months. The majority of both the snowbirds and the migrant farmworker populations exit during the hottest months, and consequently the population in Yuma is cut in half during the summer months.

5. Environment

Strengths

- The environment at NAU is well suited to support this study

Weaknesses

- None noted

Protections for Human Subjects:
Acceptable Risks and/or Adequate Protections
Relevant points of consideration of human subjects projections appear adequate for the project. Data and Safety Monitoring Plan (Applicable for Clinical Trials Only):
Not Applicable (No Clinical Trials)
Inclusion of Women, Minorities and Children: Sex/Gender: Distribution justified scientifically
Race/Ethnicity: Distribution justified scientifically
For NIH-Defined Phase III trials, Plans for valid design and analysis: Inclusion/Exclusion of Children under 18: Including ages <18; not justified scientifically
Reasonable considerations for inclusion/exclusion are provided.
Vertebrate Animals:
Not Applicable (No Vertebrate Animals)
Biohazards:
Not Applicable (No Biohazards)
Select Agents:
Not Applicable (No Select Agents)
Additional Review
Considerations Resource
Sharing Plans: Acceptable
Authentication of Key Biological and/or Chemical Resources:
Not Applicable (No Relevant Resources)
Budget and Period of Support:
Recommend as Requested

Reviewer #2
Significance: 2
Investigators: 5
Innovation: 2
Approach: 5
Environment: 4

1. **Significance**

Strengths

- The project investigates the question "Do Pathogen Genotypes, Carriage, and Social Network Differences Lead to Health Disparities in MRSA/MSSA Infections?". The goal is to determine whether national trends in infection rates and asymptomatic carriage are reflected within different ethnic groups in Yuma, Arizona; to determine if clinical strains are representative of community-carriage strains and not due to the emergence of a few, highly fit lineages; and to determine the role that social relationships and interactions have on *S. aureus* transmission.
- The project addresses an important public health concern given the mortality associated with MRSA and MSSA infections. These infections are common, with about one third of the healthy US population thought to be asymptomatic carriers of *S. aureus* and 1.5% being carriers of MRSA. Infection rates are particularly high among American Indian populations, and are lower among Hispanic populations, which relates to health equity objectives of the Collaborative.
- Differences among communities are not well understood and community-based infections are a growing public health concern.
- The proposed project meets a health priority area for the community; Yuma County Health District has identified MRSA as one of the top five priorities for infectious disease control in their latest Community Health Improvement Plan.

Weaknesses

- The potential for selection bias (resulting from the community-based events/locations of the sampling) and confounding is a concern that limits the inference that can be drawn.

  Response 10: See response 2 and response 4.

2. **Investigators**

Strengths

- Investigators have appropriate expertise in necessary disciplines including statistics, questionnaire development, microbiology, and bioinformatics.

Weaknesses

- Expertise from an infectious disease clinician, hospital/clinical infection control specialist, and infectious disease epidemiologist are lacking from the team.

  Response 11: See response 5 about our inclusion and ongoing collaborations with infectious disease specialists both in Flagstaff and in Yuma

3. **Innovation**

Strengths

- Social network analysis, coupled with more traditional microbiologic studies, is a novel combination of approaches to address the project aims.
- Investigation of ethnicity by type of residential status is innovative in assessing community-level disease patterns.

Weaknesses

- Alternative approaches regarding transmission dynamics modeling are not addressed.

  Response 12: We are not attempting to sample or model an entire outbreak, but rather gather evidence of transmission between individuals in social groups. We are not entirely sure what is meant by this comment. We eventually hope to create models of transmission dynamics that include both phylogentic and social contact variables, and

would assume that the data from this study would be highly valuable for those modeling processes. At that point we will address both systems dynamics and agent based modeling parameters, as well as alternative transmission dynamics modeling, but it seems premature to jump from the hypotheses and mechanisms that will be addressed in this study to describing alternative models of transmission dynamics.

4. **Approach**

Strengths

- The general methods are appropriate to meet the project objectives: to gain further insights into important components of *S. aureus* transmission: community carriage, pathogen genotypes, and the impact of social interactions.

Weaknesses

- Methods to investigate sex as biologic variable are not discussed.
  Response 13: See response 4 One of the key mediating or moderating variables that we will test is differences in both carriage and in levels of social contact based on sex as a biologic characteristics. In addition to sex as a biologic characteristic, gender as a cultural characteristic will be important to our analysis.

- The investigators hypothesize that pathogen populations do not differ among groups seen in clinical settings versus community settings. In order to demonstrate that the populations "do not differ", they will need to specify methods that are appropriate for equivalency analyses, based on a pre-specified margin of equivalence. This type of approach is not evident in the application.
  Response 14: We believe that the reviewer is suggesting the use of a non-inferiority or equivalence approach. To appropriately complete this type of analysis, we would need a much larger sample size to maintain the level of statistical power as determined in the sample size estimations. Rather, this must be done in a phylogenetic context that we discuss in section SA#1-7. In short, we will use Bayesian Tip-Significance testing to determine if there are any associations between phylogenetic clades and independent variables such as ethnic group or clinical vs. community isolates.

- Alternative approaches regarding transmission dynamics modeling, and infectious disease epidemiology approaches, are not addressed.
  Response 15: This appears to be identical to an earlier statement, please see See response 12

- Recruitment of study participants will occur at multiple public spaces and events. Methods to minimize selection bias are not specified. It is unclear if these sampled individuals will be representative of the larger community, for example, elderly frail individuals may be under sampled, and if the associations between exposures and infection status will be unbiased. Although the investigators note sampling by calendar month to avoid selection bias, they do not comment on the potential for selection bias due to the mechanism of contacting individuals (i.e., location of sampling).
  Response 16: See response 2

- Analysis of historical (hospital-based samples) records raises concerns of selection bias. Adjustment for factors such as age, sex, and co-morbidities are not described.
  Response 17: The hospital based samples are an important comparative baseline data set to compare with the population based data. The hospital data provides a clinical comparison within a population health context. The issue of sample bias (or actually sample difference) will be addressed in several ways, including adjustement for such factors of age, sex, and co-morbidities, which will be obtained for all of our samples.

- The sample size calculation for Aim 1 does not adjust for age as a confounding factor, which may be an important confounder given the influx of "snow birds".
  Response 18: Yes, we expect levels of colonization to be greater in persons >50 years of age. For our sample size calculation, we used a carriage rate slightly less than empirical measures of household contacts. We also used a very conservative estimation of transmission involving non-outside contacts (>0%) as work such as what we propose has not been done before. We expect that >30% of our sample population will include household contacts. This leads to an overall transmission rate of >15% which was used for our sample size calculation. Considering "snowbirds" specifically will increase the number of transmission events that we sample and will increase our power. Our use of general transmission rates therefore serves as a more conservative estimation of the number of participants needed.

- The use of logistic regression modeling does not account for the clustered observations within common social and family networks. The correlation among these sub-cluster observations is not accounted for in the analysis and is important to avoid biased estimation.
  Response 19: The use of a mixed effects logistic regression model (adding a random effect to the previously described fixed effects model) will account for the possible correlation of participants within family networks.

5. Environment

Strengths

- The basic science and statistical infrastructure and related core facilities are sufficient to support the work of the project.

Weaknesses

- The environmental support does not appear to include close interaction with clinical infectious disease expertise, which is important for the proposed sampling, analysis methods, and inference.
  Response 20: See response 5.

Protections for Human Subjects:
Acceptable Risks and/or Adequate Protections Methods to minimize risk are appropriate
Data and Safety Monitoring Plan (Applicable for Clinical Trials Only): Not Applicable (No Clinical Trials)

Inclusion of Women, Minorities and Children: Sex/Gender: Distribution justified scientifically Race/Ethnicity: Distribution justified scientifically
For NIH-Defined Phase III trials, Plans for valid design and analysis: Not applicable
Inclusion/Exclusion of Children under 18: Including ages <18; justified scientifically
Demographic groups are not excluded in the proposed sample and will include both adults and children.

Vertebrate Animals:
Not Applicable (No Vertebrate Animals)

Biohazards:
Not Applicable (No Biohazards)

Select Agents:
Not Applicable (No Select Agents)

Additional Review Considerations Resource Sharing Plans: Acceptable
Methods for data sharing are appropriate

Authentication of Key Biological and/or Chemical Resources:
Not Applicable (No Relevant Resources)

Budget and Period of Support:
Recommend as Requested

Reviewer #3

Significance: 3
Investigators: 4
Innovation: 3
Approach: 4
Environment: 2

1. Significance

Strengths

- About one third of the healthy US population is thought to be asymptomatic carriers of *S. aureus*, gram-positive bacteria and cause mortality in 20% of populations because of these infections.
- Performing a community-based study to address this infection is highly significant.
- Addressing this public health issue is important.

Weaknesses

- None noted.

2. Investigators

Strengths

- Dr. Pearson will lead this project and he is a Research Assistant Professor of Biology at NAU. He has sufficient effort and protected time to perform the studies.
- He is supported by several coinvestigators and a research technician.
- The collaborators are appropriate and well qualified.

Weaknesses

- None noted.

3. Innovation

Strengths

- Not adhering to the common paradigm of working from the hospital down, but instead from the community up is novel.

- Understanding social contacts has been critical in the control of sexually transmitted diseases, but has not been widely applied to other infectious diseases. Thus, it is somewhat innovative.

Weaknesses

- Some of the approaches are standard but appropriate.

Response 21: We agree, these methods have been validated in many different situations and we are confident that they will succeed here. Please see response 8 for further discussion.

4. Approach

Strengths

- This project proposes to define and contrast *S. aureus* carriage and circulating genotypes with clinical genotypes and infection prevalence and use genomic based phylogenetic hypotheses overlaid with defined and quantified social contact to better understand how social interactions impact transmission.
- Using genomic-based phylogenetic hypotheses overlaid with defined and quantified social contact to better understand how social interactions impact transmission and ultimately guide intervention models to limit transmission.
- Methodological approaches are well described.

Weaknesses

- Potential problems and alternative strategies are not presented.

Response 22: Many of these strategies are well tested and present little potential of failure. There are however certain threats that we discuss in sections SA#1-8 and SA#2-12. In section SA#1-8, we primarily discuss how we plan on circumventing potential difficulties in recruiting a broad cross section of the population. We discuss the impact of cultural sensitivities, recruiting from hard to reach portions of the population, sampling at multiple venues to facilitate capturing a diverse array of social interactions across ethnicities, and budgeting for additional sampling efforts. For Aim2, the primary threat, as discussed in SA#2-12 is a low incidence rate of MRSA which may reduce the observed number of genomic-based transmission events and hence, our power to identify variables most strongly associated with transmission. To mitigate this threat, we will swab three body sites, collect MRSA *and* MSSA isolates (with an expected population frequency of >30%) and characterize multiple isolates from each site.

5. Environment

Strengths

- The scientific environment and other physical resources available at NAU and the Regional Center for Border Health are excellent.

Weaknesses

- None noted

Protections for Human Subjects:
Acceptable Risks and/or Adequate Protections Data and Safety Monitoring Plan (Applicable for Clinical Trials Only):
Inclusion of Women, Minorities and Children: Sex/Gender: Distribution justified scientifically Race/Ethnicity: Distribution justified scientifically
For NIH-Defined Phase III trials, Plans for valid design and analysis:
Inclusion/Exclusion of Children under 18: Excluding ages <18; justified scientifically
Additional Review Considerations Budget and Period of Support: Recommend as Requested

Reviewer #4

Significance: 3
Investigators: 2
Innovation: 2
Approach: 1
Environment: 1

1. **Significance**

Strengths

- *S. aureus* infections, particularly MRSA can contribute to a significant health care cost.
- Data has shown a disparity in those most susceptible to infections.
- This project will examine "carriers" in the community and whether there are any racial or ethnic differences.

Weaknesses

- None noted.

2. **Investigators**

Strengths

- There is an experienced health care team.
- The PI, Dr. Reason, is a new investigator while his co-PI, Dr. Trotter, is experienced.
- The team assembled ensures success; it includes members who will help with study design, analysis, evaluation, mentoring.

Weaknesses

- None noted.

3. **Innovation**

Strengths

- This project links bacterial pathogen genome based epidemiology with social contact analysis.

Weaknesses

- None noted.

4. **Approach**

Strengths

- The study will be conducted in an ideal environment for this type of research, Yuma County, where there are two ethnic groups with different cultural behavior patterns within and between groups.

Weaknesses

- None noted.

5. **Environment**

Strengths

- Ideal environment with community and institutional support.

Weaknesses

- None noted.

Protections for Human Subjects:

Acceptable Risks and/or Adequate Protections Data and Safety Monitoring Plan (Applicable for Clinical Trials Only):

Inclusion of Women, Minorities and Children: Sex/Gender: Distribution justified scientifically
Race/Ethnicity: Distribution justified scientifically
For NIH-Defined Phase III trials, Plans for valid design and analysis: Inclusion/Exclusion of Children under 18:

Vertebrate Animals:
Not Applicable (No Vertebrate Animals)

Biohazards:
Not Applicable (No Biohazards)

Select Agents:
Not Applicable (No Select Agents)

Additional Review Considerations

Authentication of Key Biological and/or Chemical Resources:
Not Applicable (No Relevant Resources)

Budget and Period of Support:
Recommend as Requested
